# Organ system heterogeneity DB: a database for the visualization of phenotypes at the organ system level

**DOI:** 10.1093/nar/gku948

**Published:** 2014-10-13

**Authors:** Deepthi Mannil, Ingo Vogt, Jeanette Prinz, Monica Campillos

**Affiliations:** 1 German Center for Diabetes Research, Neuherberg 85764, Germany; 2 Institute of Bioinformatics and Systems Biology, Helmholtz Zentrum München, Neuherberg 85764, Germany

## Abstract

Perturbations of mammalian organisms including diseases, drug treatments and gene perturbations in mice affect organ systems differently. Some perturbations impair relatively few organ systems while others lead to highly heterogeneous or systemic effects. Organ System Heterogeneity DB (http://mips.helmholtz-muenchen.de/Organ_System_Heterogeneity/) provides information on the phenotypic effects of 4865 human diseases, 1667 drugs and 5361 genetically modified mouse models on 26 different organ systems. Disease symptoms, drug side effects and mouse phenotypes are mapped to the System Organ Class (SOC) level of the Medical Dictionary of Regulatory Activities (MedDRA). Then, the organ system heterogeneity value, a measurement of the systemic impact of a perturbation, is calculated from the relative frequency of phenotypic features across all SOCs. For perturbations of interest, the database displays the distribution of phenotypic effects across organ systems along with the heterogeneity value and the distance between organ system distributions. In this way, it allows, in an easy and comprehensible fashion, the comparison of the phenotypic organ system distributions of diseases, drugs and their corresponding genetically modified mouse models of associated disease genes and drug targets. The Organ System Heterogeneity DB is thus a platform for the visualization and comparison of organ system level phenotypic effects of drugs, diseases and genes.

## INTRODUCTION

Human diseases, drug treatments and genetically modified mouse models are perturbations in mammalian organisms with observable phenotypes. Numerous comparative analyses of these phenotypes have demonstrated that organismal phenotypes are a rich source of molecular and clinical information. Side effect similarity has been employed to identify new drug targets (1) and functional relations between disease genes have been found among diseases that share symptoms (2,3). The comparison of phenotypic information across species and perturbations has also provided novel molecular information of drugs and diseases. For example, the comparison of phenotypes of human diseases and drugs with those of genetically modified mouse models has been exploited for gene prioritization in diseases (4–6) and to predict novel drug–target interactions (7), respectively. Moreover, a semantic similarity method detecting phenotypically similar drug–disease pairs has been successful in capturing novel clinical relationships, such as contraindications (8).

**Figure 1.**
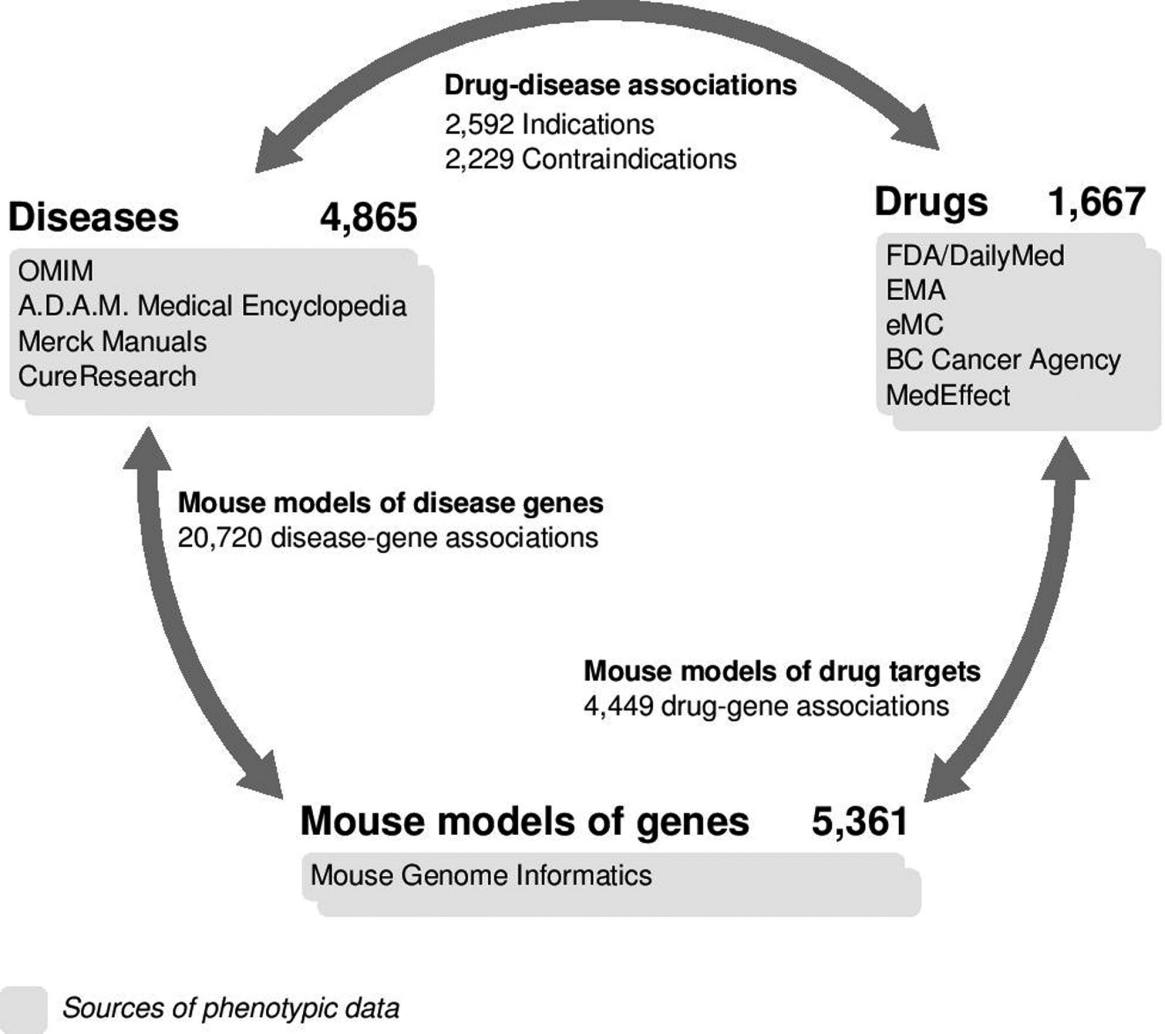
Overview of Organ System Heterogeneity DB. The number of diseases, drugs and genes with phenotypes in the database along with the number of known associations among them and the sources of phenotype data is shown. The database offers the possibility to easily compare the organ system phenotypes between the different types of perturbations.

Besides, the analysis of organismal phenotypes has evidenced the great variability of the phenotypic impact of mammalian perturbations. While some perturbations exert local effects impairing predominantly few organ systems, others cause heterogeneous effects across many organ systems, leading to a systemic harm of the organism (9,10). In a recent study (9), we analyzed the systemic impact of a large number of human diseases, drugs and genetic perturbations in mice by using the organ system heterogeneity, a measurement of the spread of phenotypic effects across multiple mammalian organ systems. We discovered a close relationship of gene properties, such as subcellular localization of the gene products, tissue expression, essentiality and the number of genes, involved in a perturbation with its systemic impact (9). This finding highlights the relevance of the analysis of phenotypic data at the organ system level for the understanding of the molecular causes linked to systemic effects of perturbations. Towards this aim, we have developed the Organ System Heterogeneity DB. This database provides the organ system level impact of disease symptoms, drug side effects and phenotypes of genetically modified mouse models. In particular, it allows the visualization of the phenotypic effects of 4865 diseases, 1667 drugs and 5361 genes (Figure 1) on 26 different organ system categories (as defined by the Medical Dictionary of Regulatory Activities (MedDRA)).

Other existing platforms, such as PhenomicDB (11), PhenomeNet (12) and PhenoHM (13), allow the extraction and comparison of specific phenotypes and genotypes across multiple species. In contrast, Organ System Heterogeneity DB provides a consolidated view on the phenotypic effects at the organ system level of perturbations, assisting in the understanding of the systemic effects of perturbations. A unique feature of Organ System Heterogeneity DB is the possibility of comparing the organ system effects of known related perturbations in a user-friendly fashion. For example, the user can compare the systemic effects of a drug with those of genetically perturbed mouse models of its known targets, and its contraindicated or indicated diseases by matching up their organ system distribution plots, their organ system heterogeneity values and the distance between their organ system distributions. Similarly, it enables the comparison of a list of perturbations according to the user's choice as well as the retrieval and comparison of the perturbations with the most similar organ system distribution to a user query. These features aid in the generation of hypotheses on new drug targets and disease genes, paving the path to improve disease treatments.

## DATABASE CONTENT

### Phenotype data

#### Distribution of phenotypic features in organ system and High Level Term (HLT) classes

**Figure 2.**
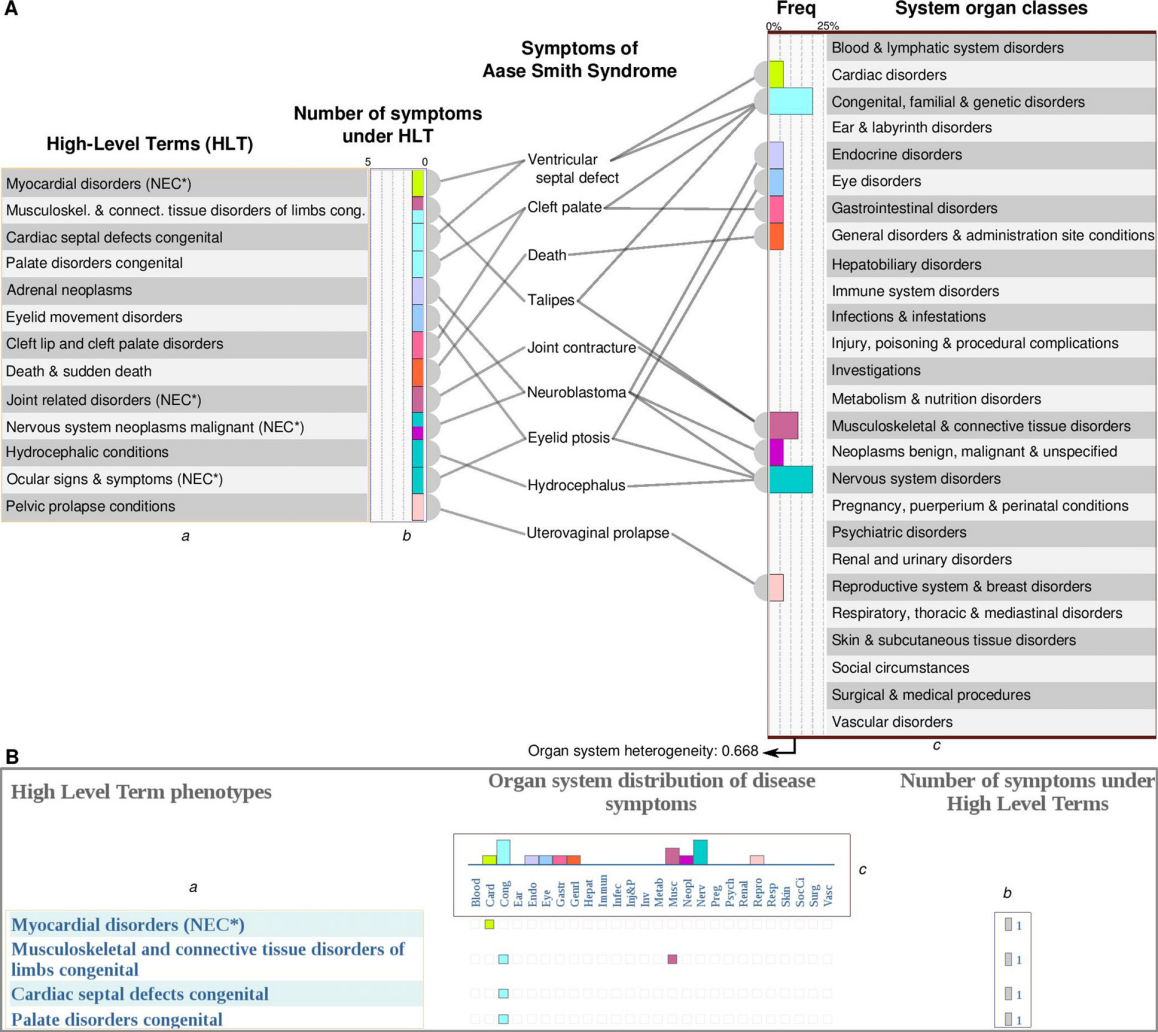
Representation and depiction of phenotype information at SOC and HLT level. (**A**) We used the hierarchical information of the MedDRA ontology to map all phenotypic features of a drug, disease or gene to the HLT and SOC level. As an example, we show the mapping of symptoms of the disease *Aase Smith syndrome* to HLT and SOC levels. After the mapping, the relative frequencies of phenotypes under each SOC and the number of phenotypes under each HLT is shown. (**B**) Screenshot of the ‘View High Level Term phenotypes’ result page of the *Aase Smith syndrome*. The phenotypes of the disease at the HLT level, the number of phenotypic traits under the HLT level phenotypes and the corresponding SOCs (indicated by colored squares) are reported. The corresponding parts of (A) and (B) are indicated as ‘*a*’ (HLT), ‘*b*’ (Number of Phenotypic features under HLT) and ‘*c*’ (SOC).

We made use of the hierarchical structure of MedDRA (http://www.meddra.org/) to annotate the phenotypic data (disease symptoms, drug side effects and phenotypes of genetic perturbations in mice) at two different levels of granularity, the System Organ Class (SOC) and the HLT level (Figure 2). MedDRA, the Medical Dictionary for Regulatory Activities terminology, is the international medical terminology developed under the auspices of the International Conference on Harmonisation of Technical Requirements for Registration of Pharmaceuticals for Human Use (ICH). MedDRA trademark is owned by IFPMA on behalf of ICH. The SOC level of MedDRA is the most general and groups all terms according to manifestation site or etiology (e.g. infections) into 26 categories. The HLT level is the most specific aggregation level that groups phenotypic features based upon anatomy, pathology, physiology, etiology or function. As depicted in Figure 2A, the mappings of the phenotypic features to the SOC level are used to derive the organ system distribution plots as well as the organ system heterogeneity value. If desired, the user can inspect the more specific phenotype information at the HLT level in relation to the organ system distribution. These distribution plots are shown in the website along with the number of the annotated phenotypic features (Figure 2B).

#### Extraction of phenotypic data

The Organ System Heterogeneity DB is based on a data set of symptoms of 4865 human diseases, side effects of 1667 drugs and phenotypes of 5361 mouse models of gene perturbations annotated previously using a MedDRA-based dictionary (8,9). In the following paragraphs we describe briefly the extraction of phenotypes for the three entities (see Figure 1 for an overview).

##### Disease symptoms

Disease phenotype data was extracted from Online Mendelian Inheritance in Man (OMIM) (14) clinical synopses as well as the following web resources: ‘The Merck Manual of Diagnosis and Therapy’ and ‘The Merck Manual Home Health Handbook’, ‘A.D.A.M. Medical Encyclopedia’ via MedlinePlus and ‘CureResearch’ (8). In total, we collected signs and symptoms coded with a MedDRA-based dictionary for 4865 diseases.

##### Drug side effects

Following the procedure used for the creation of the SIDER database (15), we parsed the phenotype data of drugs from public documents directed at health care professionals or the public reporting adverse drug events. The source documents consisted of drug labels, monographs or assessment reports published by the U.S. Food and Drug Administration (provided by FDA and DailyMed), the Medicines and Healthcare products Regulatory Agency (UK), BC Cancer Agency (Canada), MedEffect (only clinical report data, Canada) and the European Medicines Agency (8). Altogether, we obtained MedDRA-coded side effect data for 1667 drugs.

##### Phenotypes of mouse models

We extracted the phenotype annotations of mouse models encoded with the mammalian phenotype ontology (MPO) (16) provided by Mouse Genome Informatics (17) and mapped them to terms in our MedDRA-based dictionary (9). To that aim, the MPO terms were first mapped to the Unified Medical Language System (UMLS) with the help of MetaMap ( http://mmtx.nlm.nih.gov). Then, we only kept those UMLS concepts that were linked to MedDRA (9), yielding a set of 5361 mouse models of genes with phenotype data coded in MedDRA.

### Molecular and clinical relationships among drugs, diseases and genes

#### Disease genes

We collected information on genes associated to 2971 diseases resulting in 20720 disease–gene pairs in total (Figure 1). Disease genes were extracted from DisGeNET (version 2.0) (18), which integrates information from Genetic Association Database (19), Mouse Genome Database (17), OMIM (14), Comparative Toxicogenomics Database (20), PubMed and Uniprot (21). We complemented these data with information provided by MedGen (http://www.ncbi.nlm.nih.gov/books/NBK159970/), Uniprot and Orphadata (22).

#### Drug targets

We extracted direct human targets for 1002 drugs from the STITCH 3 (23) database that have a confidence score higher than 0.7 as described previously (8). In this way, 4449 drug–target pairs were obtained (Figure 1).

#### Indications and contraindications

The National Drug File-Reference Terminology (NDF-RT) (24) is an extended version of the VHA NDF and contains information on drugs approved in the United States. We obtained the public version of the NDF-RT (accessed 2 May 2012) and extracted information on indications (attributes may_prevent, may_treat and induces) and contraindications (attribute CI_with) for drugs and diseases included in our drug and disease thesaurus, respectively (8). In total, we collected 2229 drug-disease contraindications and 2592 indications (Figure 1).

### Definition of organ system heterogeneity

The organ system heterogeneity (9), a measure of the systemic impact of a perturbation, is calculated using the normalized Shannon entropy from the corresponding annotation frequencies of all SOCs and normalized by the maximum possible entropy (Equation (1)) (see Figure 2A for an example).

(1) \begin{equation*} H_{{\rm norm}_{{\rm soc}} } = - \sum\nolimits_{{\rm i} = 1}^n {\frac{{p(x_i )\log _2 (p(x_i ))}}{{\log _2 (n)}}} \end{equation*}

In Equation (1), ‘*p*(*x_i_*)’ refers to the relative annotation frequency of a SOC. ‘*n*’ equals 26, the number of different SOCs. This formula evaluates the distribution of the phenotypic effects across organ systems by accounting for the relative abundance of phenotypes. Low heterogeneity values correspond to perturbations influencing predominantly few organ systems (0 if only one organ system is affected) while high values represent effects in multiple organ systems to a similar extent (1 if all organs are affected equally).

### Distance between organ system distributions

In order to calculate the similarity between organ system distributions of two perturbations, we computed the Euclidean distance between their SOC frequency values. We then compared the resulting distance between organ system distributions to known molecular and clinical relationships of these perturbations. The distance between disease–gene pairs is benchmarked using the known disease–gene molecular associations mentioned in the section ‘Disease genes’. By using the known drug–target relationships (see section ‘Drug targets’), the distance between drug–gene pairs is benchmarked. For the benchmarking of disease–drug pairs the shared genes/targets and known clinical relationships (see section ‘Indications and contraindications’) are used. The area under the curve (AUC) values of receiver operating characteristic (ROC) plots comparing the shared molecular links between disease–drug, drug–gene and disease–gene pairs were 0.67, 0.61 and 0.65, respectively. Similarly, the AUC of ROC plot comparing the common disease–drug clinical (indications and contraindications) links with their organ system distributions distance reached a value of 0.75. This revealed an enrichment of molecular and clinical links between perturbations with similar SOC profiles (low distance between organ system distributions).

## USING ORGAN SYSTEM HETEROGENEITY DB

To search in the database, the user needs to specify first the query type (‘Disease’, ‘Drug’, ‘Gene’ or ‘Multi-search’) and enter the query term. Diseases (e.g. *Asthma*) and drugs (e.g. *Paracetamol*) can be searched by using different synonym names. To identify disease and drug names we make use of comprehensive disease and drug dictionaries. The disease dictionary includes disease names from MeSH (Medical Subject Headings, 2011), OMIM (2011) or ICD-9-CM (International Classification of Diseases, Ninth Revision, Clinical Modification, 2010) (see (8) for more information). The drug dictionary integrates different sources of drug names including PubChem (25), Drugbank (26), KEGG DRUG (27), Anatomical Therapeutic Chemical classification system and Unique Ingredient Identifiers (from the FDA Substance Registration System (http://fdasis.nlm.nih.gov/srs/srs.jsp)) (versions 2011) (see (8) for more information). Genes can be searched via name (e.g. *Dopamine receptor*), gene symbol (e.g. *KDM1A*), Ensembl Gene ID (e.g. *ENSG00000004487*), Ensembl Protein ID (e.g. *ENSP00000349049*), HGNC ID (e.g. *HGNC:23663*), UniProtKB ID (e.g. *P48506*), Entrez Gene ID (e.g. *Entrez Gene:84254*) or OMIM ID (e.g. *OMIM:134370*). As a result of a search, the database will also return entities that partially match with the query term (e.g. *Q fever* when searching *Fever*).

**Figure 3.**
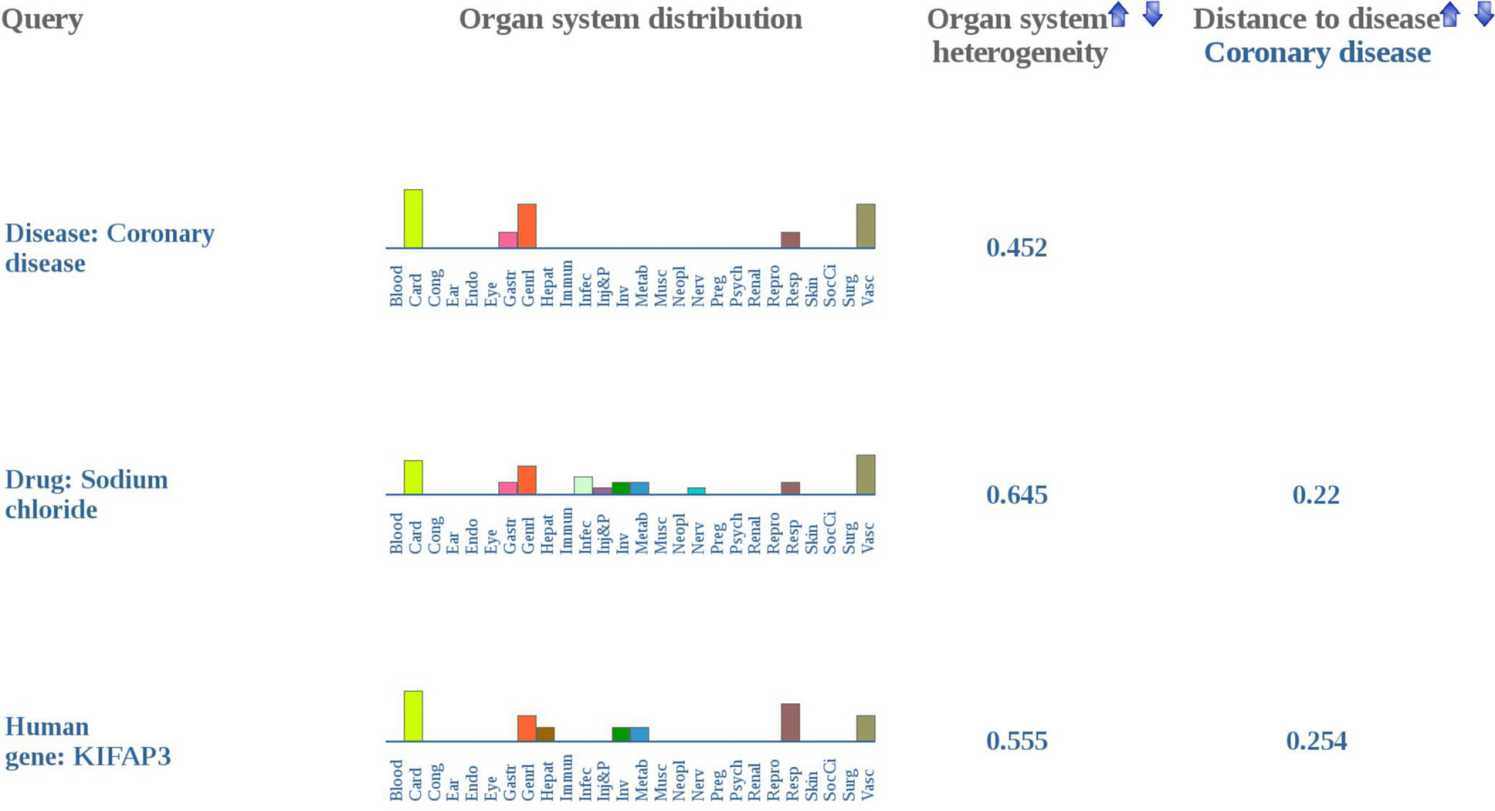
Comparison of organ system distributions via Multi-search query. Using Multi-search queries, multiple user-specified organ system distributions can be compared. The search result of the Multi-search query ‘Coronary disease|Sodium Chloride|KIFAP3’ is shown as an example.

The Multi-search option allows the inquiry of a combination of diseases, drugs or genes by separating the individual search terms with ‘|’. To reduce the complexity of the output, only exact matches of the individual search terms are returned when the Multi-search option is used. Multi-search offers the possibility to compare the organ system distributions of perturbations of the user's choice. For example, using the Multi-search query ‘Coronary disease|Sodium Chloride|KIFAP3’ (Figure 3), the disease *Coronary disease* can be compared with the drug *Sodium Chloride* and the gene coding for *Kinesin-Associated Protein 3* (*KIFAP3*). For each matching entity, the organ system distribution of the phenotypic features and the corresponding organ system heterogeneity value are displayed one below the other. For the second and subsequent matching entities the similarity distance to the first matching entity is shown. For these entities, the search results can be sorted in ascending or descending order of the organ system heterogeneity value or distance to the first entity. For an entity of interest, additional information related to its organ system distribution and more details on its phenotypes at the HLT level linked to the different organ systems can be retrieved by following the ‘Select’ link on the right.

**Figure 4.**
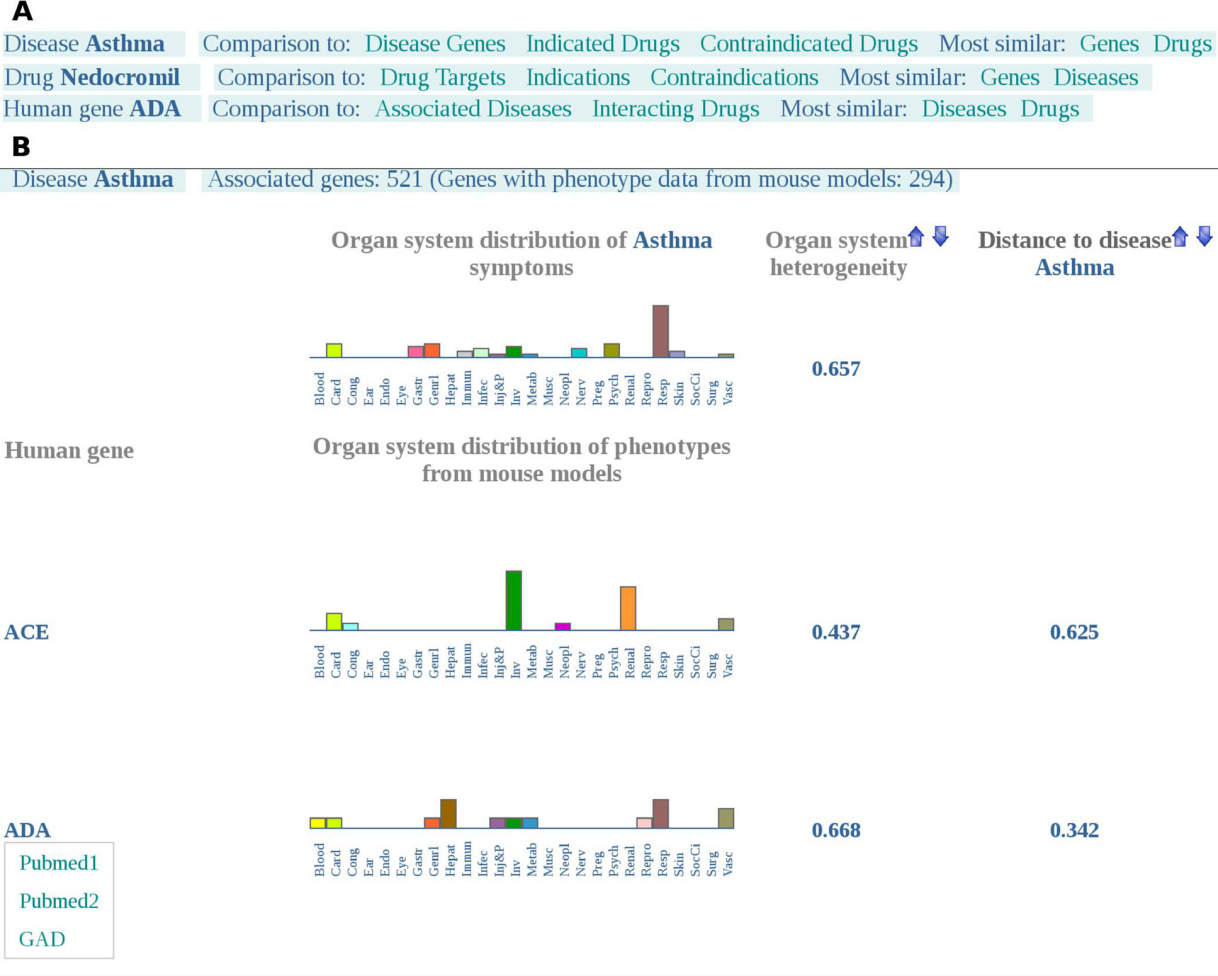
(**A**) Available options to compare organ system distributions for a given disease, drug and gene with known related entities or with entities having a similar organ system distribution. The organ system distribution of the disease *Asthma* can be compared to *Asthma*-related genes, indicated, contraindicated drugs and to genes or drugs showing the most similar organ system distribution. Phenotypic effects of drug *Nedocromil* can be compared to that of its targets, indications, contraindications, most similar genes or most similar diseases. The organ system distribution of the phenotypes resulting from perturbations in the gene *ADA* can be compared to its associated diseases, interacting drugs, most similar diseases or most similar drugs. (**B**) Comparison of organ system distributions of a disease and its associated genes. Organ system distributions of *Asthma* and two associated genes are shown. Links to different sources reporting the involvement of *ADA* in *Asthma* are available via mouse hover.

Interestingly, the database offers the possibility to compare the organ system distributions of known related entities (Figure 1) and of perturbations with similar organ system distributions. If the selected entity is a disease and information on its related disease genes and indicated or contraindicated drugs are available, its organ system distribution can be compared to that of either associated genes, indicated drugs or contraindicated drugs by clicking on ‘Disease Genes’, ‘Indicated Drugs’ or ‘Contraindicated Drugs’, respectively. Besides, by clicking on the ‘Genes’ or ‘Drugs’ option, the five genes or drugs with the shortest distance to the organ system distribution of the disease under consideration will be shown. Furthermore, the list of HLT phenotypes, the number of associated phenotypic traits, the corresponding SOCs and links to the sources of phenotypic data can be accessed by clicking on ‘View High Level Term phenotypes’ (Figure 2B). Analogously, the organ system distribution of drugs can be compared to drug targets, indications, contraindications and similar diseases and genes via the respective links. Similarly, when searching for genes, ‘Associated Diseases’, ‘Interacting Drugs’, ‘Diseases’ and ‘Drugs’ options allow the comparison with associated diseases, interacting drugs, similar diseases and drugs, respectively. Figure 4A shows the different comparison options possible for the disease *Asthma*, the drug *Nedocromil* and the gene coding for *Adenosine deaminase* (*ADA*). In the ‘Disease Genes’ web page, the sources reporting the association of a disease and a gene can be found by hovering the cursor over the gene symbol (Figure 4B). Analogously, in the ‘Associated Diseases’ web page, the sources reporting the association of a gene and a disease can be found by hovering the cursor over the disease. Following the ‘Indications’, ‘Contraindications’, ‘Indicated Drugs’ and ‘Contraindicated Drugs’ links, details on the interaction type of disease–drug associations can be found by pointing to the listed diseases or drugs. In the ‘Indications’ web page the possible interaction types are ‘Can be induced by’, ‘May be prevented by’ or/and ‘May be treated by’ and in the ‘Contraindications’ web page the interaction type is ‘Contraindication of’. In the ‘Indicated Drugs’ web page the interaction type can be ‘Induces’, ‘May prevent’ or/and ‘May treat’ and in the ‘Contraindicated Drugs’ web page the interaction type is ‘Contraindicated for’.

The comparison of organ system distributions can aid in the generation of hypothesis on novel relationships between drugs, diseases and genes. However, it should be noted that the interpretation of the similarity of organ system distributions from different types of perturbations must be taken with caution, especially when distributions of genetically modified mouse models are involved in the comparison. Although certain human diseases and the effect of many drugs on human proteins are well modeled in the mouse organism (28), the observations in mouse models cannot always be translated to human perturbations as in the case of human inflammatory diseases, which are poorly mimicked in murine models (29). This is partly due to genetic differences between the two species, such as lineage-specific gene duplication and gene loss and also due to physiological differences, which lead to specific mouse phenotypes untranslatable to a human terminology (30). Another important factor to consider when interpreting organ system distribution of different types of perturbations is the number of perturbed genes, as single gene perturbations may cause a more specific effect than drugs or diseases, which often affect several gene products (9). An additional shortcoming is that we consider an uniform degree of severity for all phenotypes. For example, the phenotypes ‘coughing and associated symptoms’ and ‘breathing abnormalities’ contribute to the organ system ‘Respiratory, thoracic and mediastinal disorders’ frequency equally, although the latter is a more severe phenotype than the former.

Despite these limitations, we have observed that perturbations with similar organ system distributions are enriched in molecular and clinical relationships (see section ‘Distance between organ system distributions’) indicating that similarity in organ system distributions can be used to infer novel relationships. This is illustrated in the example shown in Figure 3 where *Sodium Chlorid*e and *KIFAP*3 are the drug and gene, respectively, with the closest organ system distribution to *Coronary diseas*e. The relation of the *Sodium Chlorid*e with *Coronary diseas*e is strongly supported by epidemiological studies linking high levels of salt intake, which is mainly composed of *Sodium Chloride*, and cardiovascular diseases (31). Besides, the organ system distribution similarity of phenotypes from mice harboring genetically modified forms of *KIFAP3* (*Kinesin-Associated Protein 3*) and symptoms of coronary diseases may indicate the possible role of this kinesin in *Coronary disease*. In this regard, genome-wide association studies linking polymorphic forms of Kinesin 6 and ischemic heart disease and myocardial infarction point to the role of kinesin family members in the development of coronary heart disease (32–34). In summary, the Organ System Heterogeneity DB is a framework for the visualization and comparison of organ system level phenotypic effects of drugs, diseases and genes that facilitates the generation of hypothesis about novel relationship between drugs, diseases and genes.

## DESIGN AND IMPLEMENTATION

To create the Organ System Heterogeneity DB we used HTML, CSS, JavaScript and Java Servlet. We employed the 5.6.13 MySQL Community Server (GPL) to store the data and Apache Tomcat/6.0.24 as web server.

The database has four types of tables: (i) ‘Dictionary type’, (ii) ‘Phenotype type’, (iii) ‘Association type’ and (iv) ‘Similarity type’. The ‘Dictionary type’ tables store the diseases, drugs and genes for which phenotypic data is available along with their synonyms and the organ system heterogeneity value. The ‘Phenotype type’ tables store the phenotypes of diseases, drugs and genes as HLT and SOC terms. The ‘Association type’ tables store the disease-associated genes, gene-associated diseases, drug targets, interacting drugs of genes, indications of drugs, contraindications of drugs, indicated drugs for diseases and contraindicated drugs for diseases. ‘Similarity type’ tables contain the distance between the organ system distributions of all the diseases, drugs and genes. If the query type is ‘Disease’, ‘Drug’ or ‘Gene’, the query term is searched in the disease dictionary, the drug dictionary or the gene dictionary table, respectively. If the query type is ‘Multi-search’, the query term is split into the subquery terms and each subquery term is searched in all the three dictionary tables for an exact match. When matches are found, the phenotypes of the matched terms are retrieved from the ‘Phenotype type’ tables to generate the organ system distribution plots of phenotypes. The known relations between entities, such as the associated genes and indicated or contraindicated drugs of diseases, the targets, indications and contraindication of drugs as well as the associated diseases and the interacting drugs of genes, are retrieved from ‘Association tables’. The distance measures between entities are retrieved from the ‘Similarity type’ tables.

## FUTURE DIRECTIONS

Currently, the database permits the comparison of organ system distributions of diseases, drugs and genes of interest and those that are known to be related or are similar in their organ system distributions. In the future, the database can be extended with the addition of gene properties influencing the systemic impact of perturbations as well as with new accessible information of phenotypes, such as the severity of the perturbations and phenotypic data from other organisms and other types of perturbations.
